# The trajectory of fatigue over time in breast cancer patients treated with chemotherapy: exploring the effect of anthracycline-based chemotherapy

**DOI:** 10.1016/j.breast.2026.104700

**Published:** 2026-01-14

**Authors:** Anneke Kastelein, Laura Kervezee, Dieuwke R. Mink van der Molen, Daniel J. Evers, Carmen C. van der Pol, Annemiek Doeksen, N.H. Chavannes, Hans Gelderblom, Jacques Neefjes, Helena M. Verkooijen, Anne M. May

**Affiliations:** a Department of Cell and Chemical Biology, ONCODE Institute, Leiden University Medical Center, 2333 ZC, Leiden, the Netherlands; b Group of Circadian Medicine, Laboratory for Neurophysiology, Department of Cellular and Chemical Biology, Leiden University Medical Center, Leiden, the Netherlands; c Division of Imaging and Oncology, University Medical Center Utrecht, the Netherlands; d ZGT Hospital Group Twente, Almelo, the Netherlands; e Department of Surgery, Alrijne Hospital, Leiderdorp, the Netherlands; f Department of Surgery, St. Antonius Hospital, Utrecht, the Netherlands; g Department of Public Health and Primary Care, Leiden University Medical Center, Leiden, the Netherlands; h Department of Medical Oncology, Leiden University Medical Center, Leiden, the Netherlands; i Imaging Division, Netherlands Cancer Institute, Amsterdam, the Netherlands; j Julius Center for Health Sciences and Primary Care, University Medical Center Utrecht, Utrecht University, Utrecht, the Netherlands; k Division of Psychosocial Research and Epidemiology, Netherlands Cancer Institute, Amsterdam, the Netherlands

**Keywords:** Fatigue, Breast cancer, Anthracyclines, Chemotherapy, Quality of life, Cancer survivors

## Abstract

**Background:**

Cancer-related fatigue is a common and profound side effect of cancer treatment. The specific impacts of different classes of chemotherapy remains understudied. This study aimed to compare the trajectory of fatigue between breast cancer patients treated with anthracycline-based versus non-anthracycline-based regimens over a period of 4 years.

**Methods:**

Data were obtained from the Dutch UMBRELLA cohort. Included were breast cancer patients treated with (neo)adjuvant chemotherapy, categorized as anthracycline-containing vs. non-anthracycline-containing. Fatigue was measured using the Multidimensional Fatigue Inventory-20 and the fatigue scale of the EORTC QLQ-C30. A total of 1155 patients were included: 971 received anthracycline-based chemotherapy, and 184 received non-anthracycline-based chemotherapy. Linear mixed-effects models were employed to analyse time-by-treatment interactions for different dimensions of fatigue.

**Results:**

The fatigue trajectories differed significantly between treatment groups for general and physical fatigue. Pairwise comparisons indicate that anthracycline-treated patients reported higher levels of general fatigue and physical fatigue as well as reduced activity in the first six months after start of treatment. Notably, patients treated with non-anthracycline-based regimens reported better recovery over time, with significantly lower fatigue levels at 42–48 months compared to anthracyclines-treated patients, as measured by both the MFI-20 and the fatigue scale of the EORTC QLQ-C30.

**Conclusion:**

These observations suggest that (neo)adjuvant anthracycline-based chemotherapy in breast cancer patients is associated with higher levels of fatigue during treatment as well as a prolonged burden of fatigue. Treatment tailored fatigue management strategies may be particularly useful for patients undergoing anthracycline-based chemotherapy.

## Highlights

• Breast cancer patients report higher fatigue following treatment with anthracyclines than with non-anthracyclines.
• Non-anthracycline-treated patients show better long-term recovery from fatigue compared to anthracyclines-treated patients.
• Treatment tailored fatigue management may be particularly useful for patients undergoing anthracycline-based chemotherapy.

## 1 Introduction

Cancer related fatigue (CRF) is a common and disabling side-effect of cancer treatment. Cancer patients suffering from CRF experience a distressing, persistent, subjective sense of physical, emotional, and/or cognitive tiredness or exhaustion which is not proportional to recent activity, is hardly relieved by resting and interferes with usual functioning [1]. As a result, patients are often unable to regain their precancer activity level and face difficulties with daily life activities [2]. While the majority of patients recover after treatment, about a quarter of breast cancer survivors experience chronic fatigue (>6 months post-treatment) [3]. As life expectancy of cancer patients improves steadily [4], quality of life after cancer treatment becomes ever more relevant.

While different factors may play a role in the onset of CRF, studies have shown an increased risk of CRF following chemotherapy treatments [[5], [6], [7], [8], [9]]. Yet, ‘chemotherapy’ includes a wide range of different drugs. While chemotherapy as a treatment class has been shown to influence fatigue, the role of specific agents in the development of CRF are not well studied. Preclinical studies suggest that different types of chemotherapy affect fatigue to a greater or lesser extent, with the anthracycline doxorubicin (Adriamycin) having a particularly detrimental effect [[10], [11], [12]]. Doxorubicin belongs to the anthracycline drug family and is commonly used to treat breast cancer, bladder cancer, lymphoma, leukaemia, lung cancer and osteosarcoma. Recent preclinical work showed that animals treated with doxorubicin display a severely fatigued phenotype, while animals treated with other cytostatics showed recovered quickly after treatment [12]. Hence, the type of drug, and specifically doxorubicin, may have a profound influence on the development of fatigue. To date, mixed results have been reported on the specific impact of anthracycline regimens on fatigue in breast cancer survivors [[13], [14], [15], [16]]. Thus far, studies reported on a limited range of follow-up time of 28 days to 12 months and did not distinguish between the general, physical and mental dimensions of fatigue [[13], [14], [15], [16]]. Given the chronic nature of CRF, to guide supportive care, it remains crucial to evaluate the long-term trajectory of fatigue and discern any pronounced differences between types of fatigue resulting from anthracycline-based and non-anthracycline-based regimens.

The aim of this study was to compare the trajectory of fatigue over four years between breast cancer patients treated with anthracycline-based chemotherapy and non-anthracycline-based chemotherapy regimens.

## 2 Methods

Data of the present study were obtained from the UMBRELLA (Utrecht cohort for Multiple BREast cancer intervention studies and Long-term evaLuAtion) breast cancer cohort [17]. UMBRELLA was approved by the Medical Ethics Committee of the University Medical Center (UMC), Utrecht, the Netherlands, and is registered on clinicaltrials.gov (NCT02839863). Methods of data collection have previously been described extensively [17]. In summary, UMBRELLA started patient enrolment in 2013. Patients with invasive breast cancer and ductal carcinoma in situ (DCIS), aged over 18 years, and a good understanding of the Dutch language were eligible for participation in this prospective observational cohort. Informed consent was obtained for the majority of patients prior to the start of radiotherapy, and for a minority prior to surgery or during neoadjuvant systemic treatment. Clinical data and patient-reported outcomes (PROs) are being collected at regular intervals for all participants. For this study, we identified all patients who were enrolled between October 2013 and April 2022 and received chemotherapy. We included questionnaire responses up to 48 months after start of chemotherapy treatment. Male patients were excluded, as well as participants previously diagnosed with cancer, participants diagnosed with clinical metastasis (M1) or possible metastasis (Mx), pathological tumour stage 4, as well as underweight patients (BMI<18.5 kg/m^2^). Participants for whom type of chemotherapy was unknown were removed prior to analysis.

### 2.1 Data collection

Patient, tumour, and treatment characteristics were provided by the Netherlands Cancer Registry (NCR) of the Netherlands Comprehensive Cancer Organization (IKNL) [4]. Here, clinical data were collected from medical charts according to standardized procedures of trained data managers of IKNL. Data on PROs were collected through self-reported questionnaires. Eligible patients questionnaires upon inclusion, at three months, six months, and every six months thereafter. Patients were categorized according to type of chemotherapy received (i.e., anthracycline-containing and non-anthracycline containing regimes) to determine differences in fatigue. We used the fatigue questionnaire that was completed before start of chemotherapy: i.e., completion date of the questionnaire was before the start date of (neo)adjuvant chemotherapy to define timepoint 0. Scores after start of chemotherapy were collected in appropriate time bins (0–6 months, 6–12 months, etc). As such, fatigue scores are analysed relatively to start of chemotherapy rather than study inclusion date.

### 2.2 Fatigue scores

Fatigue was assessed through the Multidimensional Fatigue Inventory-20 (MFI) questionnaire. The MFI is a 20-item self-report instrument designed to measure fatigue and has been validated in cancer patients [18]. It covers five dimensions of fatigue: General Fatigue, Physical Fatigue, Mental Fatigue, Reduced Motivation and Reduced Activity. The questionnaire has an equal number of positively and negatively worded items that are rated on a 5-point Likert scale (e.g., “I feel tired”, “I feel rested”). Each dimension consists of 4 items, with the sum of scores from each dimension ranging from 4 to 20. A higher score indicates a higher level of fatigue. For none of the domains clinical cut-offs have been defined for cancer patients.

In addition, a unidimensional fatigue score was derived from the EORTC QLQ-C30 questionnaire using the fatigue symptom (FA) scale (item numbers 10, 12, 18). A higher score indicates a higher level of fatigue. The MFI-20 was provided at cohort entry, 6 months, and every six months thereafter, while the EORTC QLQ-C30 was also provided at 3 months after cohort entry.

When available, height and weight were taken from NCR data to calculate BMI. For participants for whom this NCR data was not available, height and weight were derived from self-report questionnaires. Educational level was derived from self-report questionnaires. Age was defined as age upon inclusion and was taken from NCR data.

### 2.3 Statistics

Linear mixed-effects models were used to determine the time-by-treatment (anthracycline based regimen versus no anthracycline based regimen) interaction for the different subscales of fatigue. Linear mixed-effects models were chosen as they are well suited for analysis of repeated-measures data with unequal follow-up. The models included a participant-specific random effect. Age and BMI were included as fixed effects to adjust for potential confounding as both have been shown to affect fatigue [19,20]. Changes in self-reported fatigue are presented as the estimated marginal means with standard errors (SE), reflecting the difference between patients treated with anthracycline-based chemotherapy and non-anthracycline-based chemotherapy. For post-hoc testing a pairwise approach comparing the estimated marginal means was chosen. A value of p < 0.05 was deemed significant, and given the exploratory nature of this analysis, no p-value adjustment has been applied. The standardised effect size (ES) was measured by Cohen's d (observed difference divided by the population standard deviation). To contextualise fatigue levels in our cohort, MFI and FA scores were compared with published age- and sex-matched population sample reference values derived from Germany [21] and the Netherlands [22] respectively.

To capture potential nonlinear relationships in the data, we attempted to incorporate splines into our linear mixed-effects model. Nevertheless, the linear mixed-effects model without splines, provided a better overall fit and was thus used for subsequent analysis.

Analyses were performed using R version 4.3.1, R studio version 2023.06.0. Statistical analysis packages used included emmeans [23], splines [24] and lmerTest [25].

## 3 Results

### 3.1 Patients

**Fig. 1:**
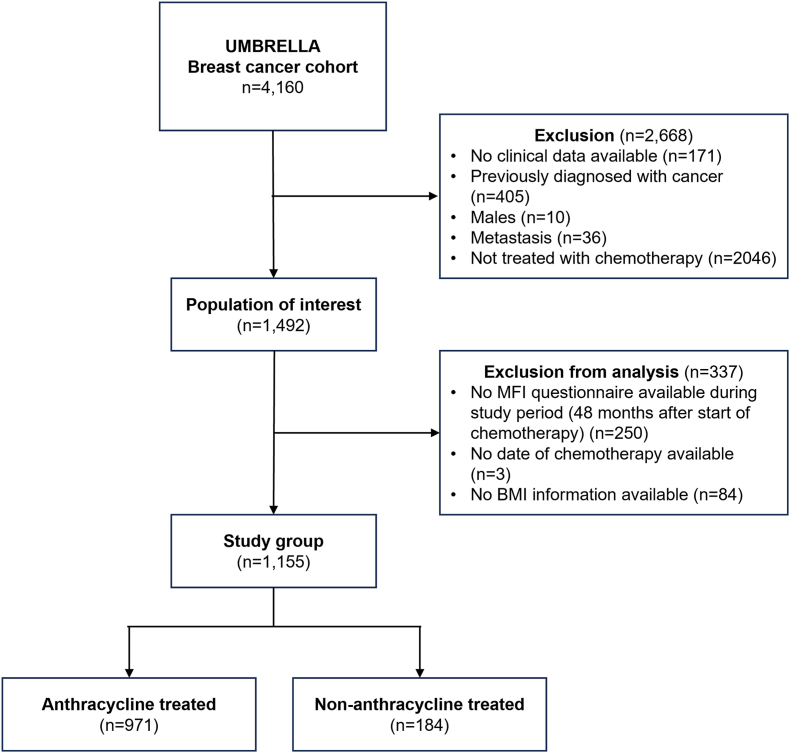
Flowchart of patient inclusion within the UMBRELLA cohort between October 2013 and April 2022.

In total, 1155 patients were included in the analysis: 971 of them were treated with anthracyclines, and 184 patients were treated with non-anthracycline based therapy (Fig. 1). Patient characteristics at baseline are shown in Table 1. Of note, patients treated with anthracyclines more often presented with a higher pathological tumour stage (T2) and more often received endocrine treatment.

**Table 1:** Demographics of included participants from the UMBRELLA cohort, treated with anthracycline or non-anthracycline based chemotherapy.

Characteristic	Anthracyclines, N = 971^a^	No anthracyclines, N = 184^a^
Age	51 (45, 58)	53 (48, 61)
Radiotherapy	908 (94 %)	162 (88 %)
Endocrine treatment	663 (68 %)	102 (55 %)
Menopausal status at inclusion		
Perimenopausal	69 (7.1 %)	6 (3.3 %)
Postmenopausal	340 (35 %)	71 (38.6 %)
Premenopausal	367 (37.8 %)	51 (27.7 %)
Missing	195 (20 %)	56 (30.4 %)
Pathological tumour stage		
T0	131 (13 %)	46 (25 %)
T1	434 (45 %)	87 (47 %)
T2	293 (30 %)	24 (13 %)
T3	50 (5.1 %)	4 (2.2 %)
X	37 (3.8 %)	12 (6.5 %)
Missing	26 (2.7 %)	11 (6.0 %)
BMI		
Healthy BMI (18,5–25)	432 (44 %)	85 (46 %)
Overweight (25–30)	344 (35 %)	61 (33 %)
Obese (>30)	195 (20 %)	38 (21 %)
Educational level		
No education	10 (1.0 %)	2 (1.1 %)
Low	168 (17.3 %)	34 (18.5 %)
Medium	310 (31.9 %)	54 (29.3 %)
High	472 (48.6 %)	92 (50 %)
Missing	11 (1.1 %)	2 (1.1 %)

Abbreviations: BMI: Body Mass Index, Education: no education; low (primary school or prevocational education); medium (upper secondary education and vocational training); high (applied university and university degree programs).

a Median (IQR); n (%).

### 3.2 Association between treatment with anthracyclines and different dimensions of fatigue

**Fig. 2:**
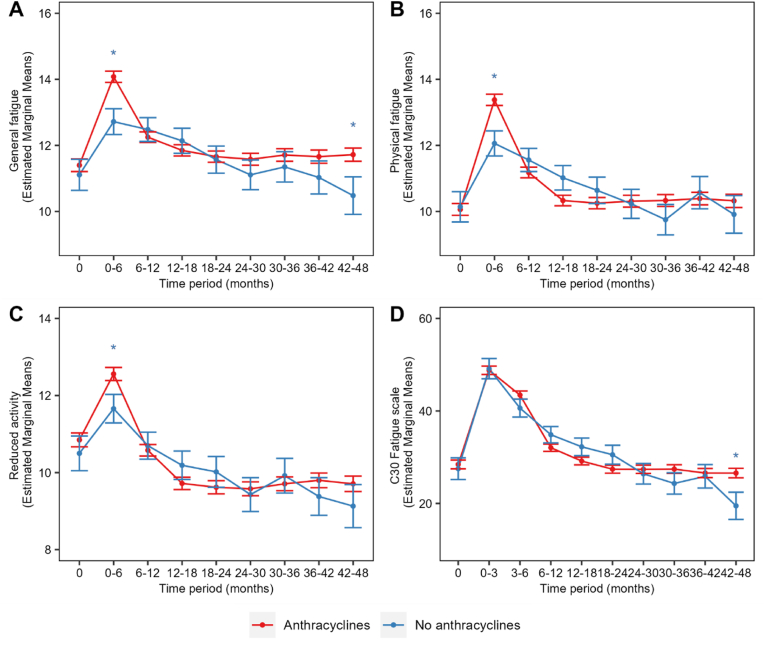
Trajectory of the fatigue per MFI subscale and EORTC QLQ-C30 fatigue symptom scale over time after anthracycline (red) or non-anthracycline (blue) based treatment: **(A)** MFI general fatigue score, **(B)** MFI physical fatigue, **(C)** MFI reduced activity **(D)** EORTC QLQ-C30 fatigue scale. Results are presented as model predicted estimated marginal means accompanied by the standard error. Lower scores indicate less fatigue. ∗p < 0.05. (For interpretation of the references to colour in this figure legend, the reader is referred to the Web version of this article.)

Both treatment groups exhibit a notable increase in general fatigue following the start of chemotherapy treatment, particularly in the group receiving anthracycline-based therapy (Fig. 2A). Compared with an age-matched population, patients in our cohort report consistently higher fatigue scores throughout the study duration (reference mean score 8.7; 75th percentile: 11) [21]. Over time, a reduction in general fatigue was observed, especially among patients receiving non-anthracycline-based therapy. Scores among anthracycline-treated patients remained above the 75th percentile of the reference population throughout the entire four-year follow-up period, indicating persistently elevated fatigue. Analysis revealed a significant interaction between type of chemotherapy (anthracycline vs non-anthracycline based therapy) and time for general fatigue (p = 0.004) indicating a difference in trajectory of fatigue over time between groups (Table 2). Pairwise comparisons between groups using the estimated marginal means (Fig. 2A) revealed that patients treated with anthracyclines reported significantly higher general fatigue (mean difference between anthracycline group and non-anthracycline group 1.35, 95 %CI:0.53–2.18, ES:0.46) during 0–6 months after onset of chemotherapy treatment (Fig. 2A). Over time patients in the non-anthracycline group showed a further decrease in general fatigue with a significant difference at 42–48 months post-treatment (mean difference 1.24, 95 %CI:0.06–2.42, ES:0.43). Estimated marginal means per scale for each time point are shown in Supplement Table 1.

**Table 2:** Test statistics for all MFI fatigue scales and EORTC C30 QLQ fatigue symptom scale.

	Sum Sq	Mean Sq	DF	DenDF	F value	p value
**General fatigue**						
Time (months)	1117.882	139.735	8	4662.263	16.406	0.000
Class of chemotherapy	16.188	16.188	1	1283.881	1.901	0.168
Age	311.433	311.433	1	1181.048	36.565	0.000
BMI category	380.133	190.067	2	1137.341	22.315	0.000
Time x Class of chemotherapy	193.230	24.154	8	4662.236	2.836	0.004
**Physical fatigue**						
Time (months)	1687.723	210.965	8	4665.682	24.696	0.000
Class of chemotherapy	0.508	0.508	1	1273.964	0.059	0.807
Age	56.102	56.102	1	1166.727	6.567	0.011
BMI category	747.494	373.747	2	1121.093	43.752	0.000
Time x Class of chemotherapy	244.151	30.519	8	4665.651	3.573	0.000
**Mental fatigue**						
Time (months)	151.570	18.946	8	4648.704	2.632	0.007
Class of chemotherapy	0.136	0.136	1	1281.729	0.019	0.891
Age	266.440	266.440	1	1184.971	37.014	0.000
BMI category	85.767	42.884	2	1143.901	5.957	0.003
Time x Class of chemotherapy	92.263	11.533	8	4648.683	1.602	0.119
**Reduced activity**						
Time (months)	1532.434	191.554	8	4673.488	22.748	0.000
Class of chemotherapy	1.687	1.687	1	1282.075	0.200	0.655
Age	3.599	3.599	1	1173.660	0.427	0.513
BMI category	459.779	229.890	2	1127.504	27.301	0.000
Time x Class of chemotherapy	126.519	15.815	8	4673.456	1.878	0.059
**Reduced motivation**						
Time (months)	394.717	49.340	8	4669.558	7.818	0.000
Class of chemotherapy	1.598	1.598	1	1281.711	0.253	0.615
Age	48.673	48.673	1	1174.903	7.712	0.006
BMI category	180.846	90.423	2	1129.455	14.328	0.000
Time x Class of chemotherapy	56.460	7.057	8	4669.527	1.118	0.347
**C30 Fatigue symptom scale**						
Time (months)	137913.458	15323.718	9	5400.209	63.171	0.000
Class of chemotherapy	42.372	42.372	1	1258.261	0.175	0.676
Age	5459.736	5459.736	1	1166.595	22.508	0.000
BMI category	5413.657	2706.828	2	1124.543	11.159	0.000
Time x Class of chemotherapy	5565.100	618.344	9	5400.110	2.549	0.006

Abbreviations: Sum Sq: sum of squares, Mean Sq: mean of the sum of squares; Df: degrees of freedom; DenDF: denominator degrees of freedom.

For physical fatigue, patients reported elevated scores compared to the general population (reference mean score: 8.2, 75th percentile: 10) [21] following the start of chemotherapy treatment, particularly when treated with anthracycline-based therapy (Fig. 2B). Over time, both groups show a decrease in fatigue scores, although the scores remain above the 75th percentile of age-matched population scores [21]. A significant interaction effect was found between time and type of chemotherapy (p < 0.001, Table 2). Post-hoc comparisons show a significant difference between treatment groups during 0–6 months of treatment (mean difference 1.32, 95 %CI:0.51–2.12, ES:0.45), no significant differences are observed between the groups at other timepoints (Fig. 2B). Similar trajectories were observed for the reduced activity subscale: both groups reported high levels of fatigue during 0–6 months of treatment that were above the reference population (≥75th percentile reference score 11, mean score 8.2) [21], but that decreased over time (Fig. 2C). The analysis indicated a non-significant interaction effect between time and chemotherapy regimen (p = 0.059, Table 2). Post-hoc analysis of estimated marginal means revealed a slight difference between the groups at 0–6 months (mean difference 0.90, 95 %CI:0.10–1.70, ES:0.31, Fig. 2C).

No significant time-by-treatment interaction was detected for the mental fatigue (p = 0.119) or reduced motivation (p = 0.347) subscales (Table 2).

### 3.3 EORTC QLQ-C30 fatigue symptom scale

For this questionnaire, an additional timepoint was available (3 months after cohort entry). The same patients were included as for the analysis of the MFI-20 scales. For both groups, fatigue scores increase after onset of treatment (0–3 months) to levels that are considerably higher than age- and sex-matched population scores (mean score: 22.8) [22] and gradually decline over time, particularly in the non-anthracycline-treated group (Fig. 2D). Our model showed a significant interaction effect of time and type of chemotherapy on fatigue (p = 0.006, Table 2). Post-hoc testing showed no significant difference early after onset of chemotherapy, however, 43–48 months after treatment the anthracycline treated group reported significantly more fatigue (mean difference = 7.09, 95 %CI:0.99–13.20, ES:0.46, Supplement Table 2, Fig. 2D).

Given the higher prevalence of endocrine therapy in the anthracycline-treated group (68 % vs. 55 %), we conducted a sensitivity analysis including an interaction term between endocrine therapy and chemotherapy class. This addition did not substantially alter the direction or magnitude of the model estimates (Supplement Table 3).

## 4 Discussion

Understanding the risk factors for CRF during and after treatment may provide opportunities for identifying high-risk patients and motivate them for effective interventions like exercise [26], as well as shed light on why some patients develop chronic fatigue while others recover. While earlier studies have compared the shorter-term effects of anthracycline versus non-anthracycline regimens, this study evaluated the effects of these regimens on different fatigue dimensions up to four years after start of treatment. After the start of chemotherapy, both treatment groups reported an increase of fatigue across domains. Anthracycline-treated patients showed substantial increases in general fatigue (on both the MFI and FA scale) and physical fatigue, exceeding the minimal clinically important difference for both instruments [27,28]. Non-anthracycline-treated patients reported smaller increases in fatigue, and the threshold for clinical important difference was reached only for the FA scale. Following this initial peak, fatigue across the different domains gradually decreased over time in both groups. Over the four-year follow-up we observed that the trajectories of general and physical fatigue are significantly different between regimens. During the treatment phase anthracycline-treated patients reported higher general and physical fatigue compared to non-anthracycline-treated patients, as well as reduced physical activity. Moreover, we found that 42 months post-treatment, patients who were not exposed to anthracyclines reported less general fatigue and less fatigue on the FA scale compared anthracycline-treated patients. While statistically significant, it should be noted that the observed differences between groups are below the threshold of two points that is considered the minimal clinically important difference for the MFI [27]. Nonetheless, the observed effect sizes (ranging from 0.31 to 0.46) fall within the small-to-moderate range and therefore suggest that anthracycline-based treatment may have a meaningful impact on fatigue levels for patients, even when the absolute score difference is modest. Compared with age-matched population norms, the elevated scores in anthracycline-treated patients indicate elevated fatigue that persist for several years after treatment [21]. For the EORTC-QLQ-30 FA scale, the difference observed at 42–48 months was on the threshold for minimal clinical significance [28]. In line with the study's objective, these findings should be interpreted as exploratory and underscore the need for further research.

The MFI-20 allowed us to disentangle the effect of anthracycline treatment on different dimensions of fatigue. Anthracycline-treated patients reported more general fatigue, physical fatigue, and reduced activity during the first 0–6 months after start of treatment compared to anthracycline-based-therapy. Thus far reports have been inconsistent on the differences between anthracycline- and non-anthracycline-treated breast cancer patients during and shortly after treatment. In 2004, de Jong and colleagues reported that anthracycline-based treatment was associated with a peak in fatigue during treatment while non-anthracycline-treated patients reported a delayed peak four weeks post-treatment [14]. Moreover, fatigue peaked higher in anthracycline-treated patients compared to those receiving a non-anthracycline regimen across two treatment cycles [13]. In 2019, researchers showed that the proportion of patients on anthracycline-based regimens reporting fatigue [15] was higher compared to non‐anthracycline‐based regimens throughout the entire course of treatment. However, a recently published study reported no differences in fatigue between anthracycline and non-anthracycline regimens 3 and 12 months after diagnosis [16]. Differences in methods, questionnaires, and follow-up duration complicate a comparison these studies. In our study, we also observed differences between questionnaires. The differences observed in general and physical fatigue 0–6 months after onset of treatment are not significant when assessed with the FA scale. These findings suggest that the choice of questionnaire may significantly impact the detection of differences between cancer patient groups. The MFI-20 questionnaire's ability to distinguish specific dimensions of fatigue, may provide a more balanced understanding of fatigue trajectories in cancer patients.

Remarkably, patients treated with non-anthracycline-based regimens appear to recover better over time in terms of general fatigue and scores on the FA scale, reporting significantly lower fatigue levels 42–48 months after treatment compared to anthracycline-based therapy. To the best of our knowledge, this is the first study to report a long-term difference in fatigue outcomes between anthracycline-treated and non-anthracycline-treated patients. These differences in chemotherapy-induced damage could be attributed to variations in drug mechanisms, dosage regimens, or individual patient factors. However, to date, no systematic comparisons have been conducted between these treatments and the extent and nature of damage may vary across different classes of chemotherapeutics. Preclinical studies suggest fatigue may be related to the mechanism of action of anthracyclines, as the combination of DNA damage and chromatin damage specifically induces fatigue [12]. Anthracyclines have been shown to induce (neuro)inflammation [29], oxidative stress and mitochondrial dysfunction [30], and neurotoxic effects [31]. These effects may affect physical and general fatigue more strongly than other fatigue dimensions and may partly explain why differences emerged in some fatigue dimensions but not others. This hypothesis warrants further investigation.

The strengths of our study include a large patient cohort, the long follow-up, and the use of validated and widely used instruments that enable the evaluation of long-term fatigue trajectories that goes beyond existing literature. The use of the MFI-20 allowed us to assess different dimensions of fatigue, which could be particularly relevant for designing treatment strategies that target specific aspects such as physical or general fatigue. A limitation of our study is that the UMBRELLA cohort was not designed to specifically evaluate the impact of chemotherapy. Patients were enrolled during their initial consultation with the radiation oncologist, and the timing of questionnaires was not aligned with chemotherapy schedules. While the use of linear mixed-effects models is well suited for the analysis of repeated measures data with unequal follow up, variation in questionnaire timing, particularly during the treatment phase, may have increased within-group variability and reduced the precision of estimates of fatigue. Moreover, the sample of non-anthracycline-treated patients was relatively small at the later timepoints, which could have impacted the robustness of our findings. Finally, as this study was conducted in a Dutch breast cancer cohort, generalisability to other populations should be interpreted with care. Nonetheless, the use of treatment regimens based on international guidelines and validated fatigue measures supports relevance to similar healthcare settings. Future studies would benefit from a prospective design, allowing for the alignment of questionnaire timing with treatment schedules and the collection of more detailed data on treatment regimens. Additional (pre)clinical research may further examine the underlying biological mechanisms of long-term fatigue resulting from anthracyclines and evaluate targeted interventions.

In conclusion, breast cancer patients treated with (neo)adjuvant anthracycline-based chemotherapy experience greater general and physical fatigue during the treatment phase compared to those receiving non-anthracycline regimens. Importantly, our findings suggest that non-anthracycline-treated patients demonstrate better long-term recovery from fatigue. These results may help clinicians anticipate fatigue more effectively, address the implications for quality of life during treatment discussions, and highlight the potential for tailored fatigue management strategies, particularly for patients undergoing anthracycline-based chemotherapy.

## CRediT authorship contribution statement

**Anneke Kastelein:** Writing – original draft, Visualization, Software, Methodology, Formal analysis, Conceptualization. **Laura Kervezee:** Writing – review & editing, Validation, Supervision. **Dieuwke R. Mink van der Molen:** Writing – review & editing, Investigation, Data curation. **Daniel J. Evers:** Writing – review & editing, Resources. **Carmen C. van der Pol:** Writing – review & editing, Resources. **Annemiek Doeksen:** Writing – review & editing, Resources. **N.H. Chavannes:** Writing – review & editing, Supervision. **Hans Gelderblom:** Writing – review & editing, Supervision. **Jacques Neefjes:** Writing – review & editing, Supervision. **Helena M. Verkooijen:** Writing – review & editing, Resources, Investigation. **Anne M. May:** Writing – review & editing, Supervision, Conceptualization.

## Ethics approval

UMBRELLA was approved by the Medical Ethics Committee of the University Medical Center (NL52651.041.15, Medical Ethics Committee 15/165), Utrecht, the Netherlands, and is registered on clinicaltrials.gov (NCT02839863).

## Funding

This research did not receive any specific grant from funding agencies in the public, commercial, or not-for-profit sectors. AK is funded through a Spinoza premium provided by The 10.13039/501100003246Dutch Research Council (NWO) to JN (SPI 93-539).

## Declaration of competing interest

The authors declare that they have no known competing financial interests or personal relationships that could have appeared to influence the work reported in this paper.

## Data Availability

The data is available on request for non-commercial scientific research, subject to study question, privacy and confidentiality restrictions, and registration.

## References

[1] Berger A.M., Mooney K., Alvarez-Perez A. (2015). Cancer-related fatigue, version 2.2015. J Natl Compr Cancer Netw.

[2] Bootsma T.I., Schellekens M.P.J.J., van Woezik R.A.M.M., van der Lee M.L., Slatman J. (2020). Experiencing and responding to chronic cancer-related fatigue: a meta-ethnography of qualitative research. Psychooncology.

[3] Abrahams H.J.G., Gielissen M.F.M., Schmits I.C., Verhagen C.A.H.H.V.M., Rovers M.M., Knoop H. (2016). Risk factors, prevalence, and course of severe fatigue after breast cancer treatment: a meta-analysis involving 12 327 breast cancer survivors. Ann Oncol.

[4] Integraal Kankercentrum Nederland The incidence, prevalence and survival data derived from the Netherlands cancer registry. https://iknl.nl/nkr-cijfers.

[5] Bower J.E., Ganz P.A., Desmond K.A., Rowland J.H., Meyerowitz B.E., Belin T.R. (2000). Fatigue in breast cancer survivors: occurrence, correlates, and impact on quality of life. J Clin Oncol.

[6] Jacobsen P.B., Donovan K.A., Small B.J., Jim H.S., Munster P.N., Andrykowski M.A. (2007). Fatigue after treatment for early stage breast cancer: a controlled comparison. Cancer.

[7] Goedendorp M.M., Andrykowski M.A., Donovan K.A. (2012). Prolonged impact of chemotherapy on fatigue in breast cancer survivors: a longitudinal comparison with radiotherapy-treated breast cancer survivors and noncancer controls. Cancer.

[8] de Ligt K.M., Heins M., Verloop J., Smorenburg C.H., Korevaar J.C., Siesling S. (2019). Patient-reported health problems and healthcare use after treatment for early-stage breast cancer. Breast.

[9] Ma Y., He B., Jiang M. (2020). Prevalence and risk factors of cancer-related fatigue: a systematic review and meta-analysis. Int J Nurs Stud Elsevier Ltd.

[10] Elsea C.R., Kneiss J.A., Wood L.J. (2015). Induction of IL-6 by cytotoxic chemotherapy is associated with loss of lean body and fat mass in tumor-free female mice. Biol Res Nurs.

[11] Zombeck J.A., Fey E.G., Lyng G.D., Sonis S.T. (2013). A clinically translatable mouse model for chemotherapy-related fatigue. Comp Med.

[12] Wang Y., van der Zanden S.Y., van Leerdam S. (2022). Induction of fatigue by specific anthracycline cancer drugs through disruption of the circadian pacemaker. Cancers.

[13] de Jong N., Candel M.J.J.M., Schouten H.C., Abu-Saad H.H., Courtens A.M. (2004). Prevalence and course of fatigue in breast cancer patients receiving adjuvant chemotherapy. Ann Oncol.

[14] De Jong N., Kester A.D.M., Schouten H.C., Abu-Saad H.H., Courtens A.M. (2006). Course of fatigue between two cycles of adjuvant chemotherapy in breast cancer patients. Cancer Nurs.

[15] Nyrop K.A., Deal A.M., Shachar S.S. (2019). Patient‐reported toxicities during chemotherapy regimens in current clinical practice for early breast cancer. Oncologist.

[16] Avis N.E., Beverly, Levine J. (2024 328. 2024). The impact of non- and anthracycline-based chemotherapy on fatigue in breast cancer survivors: results from WF-97415. Support Care Cancer.

[17] Young-Afat D.A., van Gils C.H., van den Bongard H.J.G.D. (2017). The Utrecht cohort for multiple BREast cancer intervention studies and long-term evaLuAtion (UMBRELLA): objectives, design, and baseline results. Breast Cancer Res Treat.

[18] Smets E.M.A., Garssen B., Bonke B., De Haes J.C.J.M. (1995). The multidimensional fatigue inventory (MFI) psychometric qualities of an instrument to assess fatigue. J Psychosom Res.

[19] Bischel L.E., Ritchie C., Kober K.M. (2016). Age differences in fatigue, decrements in energy, and sleep disturbance in oncology patients receiving chemotherapy. Eur J Oncol Nurs.

[20] Kastelein A., Mols F., Kervezee L. (2025). The impact of chemotherapy and body mass index on cancer-related fatigue in Colon cancer patients: a PROFILES-registry study. Cancer Med.

[21] Schwarz R., Krauss O., Hinz A. (2003). Fatigue in the general population. Onkologie.

[22] de Ligt K.M., Aaronson N.K., Liegl G., Nolte S. (2023). Updated normative data for the EORTC QLQ-C30 in the general Dutch population by age and sex: a cross-sectional panel research study. Qual Life Res.

[23] Russell V. (2023). Lenth. emmeans: estimated marginal means, Aka least-squares means. https://cran.r-project.org/package=emmeans.

[24] R Core Team (2023). https://www.r-project.org/.

[25] Kuznetsova A., Brockhoff P.B., Christensen R.H.B. (2017). lmerTest package: tests in linear mixed effects models. J Stat Software.

[26] Ligibel J.A., Bohlke K., May A.M. (2022). Exercise, diet, and weight management during cancer treatment: ASCO guideline. J Clin Oncol.

[27] Purcell A., Fleming J., Bennett S., Burmeister B., Haines T. (2010). Determining the minimal clinically important difference criteria for the multidimensional fatigue inventory in a radiotherapy population. Support Care Cancer.

[28] Musoro J.Z., Coens C., Sprangers M.A.G. (2023). Minimally important differences for interpreting EORTC QLQ-C30 change scores over time: a synthesis across 21 clinical trials involving nine different cancer types. Eur J Cancer.

[29] Borniger J.C., Ii W.H.W., Gaudier-Diaz M.M. (2017).

[30] Tacar O., Sriamornsak P., Dass C.R. (2013). Doxorubicin: an update on anticancer molecular action, toxicity and novel drug delivery systems. J Pharm Pharmacol.

[31] Du J., Zhang A., Li J. (2021). Doxorubicin-induced cognitive impairment: the mechanistic insights. Front Oncol.

