# Supplementary material for: The trajectory of fatigue over time in breast cancer patients treated with chemotherapy: exploring the effect of anthracycline-based chemotherapy
Source: Breast. 2026 Jan 14;85:104700. doi: 10.1016/j.breast.2026.104700 (PMC12856468; doi:10.1016/j.breast.2026.104700)
Supplement: Multimedia component 1 [file mmc1.docx]

**Supplementary materials**

|  |  |  | **General fatigue** | | | | | **Physical fatigue** | | | | | **Mental fatigue** | | | | | **Reduced activity** | | | | | **Reduced motivation** | | | | |
| --- | --- | --- | --- | --- | --- | --- | --- | --- | --- | --- | --- | --- | --- | --- | --- | --- | --- | --- | --- | --- | --- | --- | --- | --- | --- | --- | --- |
| **Time since onset chemotherapy (months)** | **Anthracyclines**  **(n patients)** | **No anthracyclines**  **(n patients)** | **estimate** | **SE** | **df** | **Lower CL** | **upper.CL** | **estimate** | **SE** | **df** | **lower.CL** | **upper.CL** | **estimate** | **SE** | **df** | **lower.CL** | **upper.CL** | **estimate** | **SE** | **df** | **lower.CL** | **upper.CL** | **estimate** | **SE** | **df** | **lower.CL** | **upper.CL** |
| 0 | 445 | 67 | 0.29 | 0.50 | 4476.3 | -0.69 | 1.27 | -0.08 | 0.49 | 4615.3 | -1.04 | 0.89 | 0.13 | 0.47 | 4301.9 | -0.79 | 1.05 | 0.36 | 0.49 | 4633.7 | -0.60 | 1.31 | 0.07 | 0.42 | 4590.8 | -0.76 | 0.90 |
| 0-6 | 585 | 116 | 1.35 | 0.42 | 3257.1 | 0.53 | 2.18 | 1.32 | 0.41 | 3410.3 | 0.51 | 2.12 | 0.79 | 0.40 | 3082.1 | 0.01 | 1.57 | 0.90 | 0.41 | 3431.6 | 0.10 | 1.70 | 0.56 | 0.35 | 3382.2 | -0.13 | 1.26 |
| 6-12 | 775 | 147 | -0.22 | 0.39 | 2691.0 | -0.99 | 0.54 | -0.38 | 0.38 | 2818.1 | -1.13 | 0.36 | -0.26 | 0.37 | 2550.3 | -0.98 | 0.47 | -0.12 | 0.38 | 2836.1 | -0.86 | 0.62 | -0.22 | 0.33 | 2794.6 | -0.86 | 0.43 |
| 12-18 | 678 | 121 | -0.29 | 0.41 | 3118.9 | -1.10 | 0.52 | -0.69 | 0.40 | 3261.3 | -1.49 | 0.10 | -0.49 | 0.39 | 2956.9 | -1.26 | 0.28 | -0.47 | 0.40 | 3281.1 | -1.26 | 0.31 | -0.32 | 0.35 | 3235.1 | -1.00 | 0.37 |
| 18-24 | 607 | 97 | 0.09 | 0.44 | 3623.0 | -0.78 | 0.95 | -0.39 | 0.43 | 3777.9 | -1.23 | 0.46 | -0.12 | 0.42 | 3441.2 | -0.94 | 0.70 | -0.40 | 0.43 | 3799.2 | -1.24 | 0.44 | 0.12 | 0.37 | 3749.9 | -0.62 | 0.85 |
| 24-30 | 507 | 74 | 0.48 | 0.48 | 4282.0 | -0.47 | 1.42 | 0.08 | 0.47 | 4433.3 | -0.84 | 1.01 | 0.30 | 0.45 | 4095.1 | -0.59 | 1.18 | 0.16 | 0.47 | 4453.4 | -0.76 | 1.08 | 0.04 | 0.41 | 4406.4 | -0.76 | 0.84 |
| 30-36 | 467 | 67 | 0.36 | 0.50 | 4503.9 | -0.61 | 1.34 | 0.58 | 0.49 | 4646.1 | -0.38 | 1.54 | -0.18 | 0.47 | 4324.4 | -1.09 | 0.73 | -0.21 | 0.48 | 4664.9 | -1.16 | 0.74 | 0.03 | 0.42 | 4621.1 | -0.80 | 0.85 |
| 36-42 | 399 | 54 | 0.64 | 0.53 | 4944.8 | -0.41 | 1.69 | -0.18 | 0.53 | 5059.5 | -1.21 | 0.86 | 0.23 | 0.50 | 4791.7 | -0.75 | 1.21 | 0.42 | 0.52 | 5074.1 | -0.60 | 1.45 | 0.52 | 0.45 | 5039.7 | -0.37 | 1.41 |
| 42-48 | 347 | 38 | 1.24 | 0.60 | 5400.8 | 0.06 | 2.42 | 0.41 | 0.60 | 5455.9 | -0.77 | 1.58 | -0.03 | 0.56 | 5316.8 | -1.13 | 1.08 | 0.58 | 0.59 | 5462.4 | -0.58 | 1.74 | 0.40 | 0.52 | 5446.9 | -0.61 | 1.41 |

*Supplement Table 1. Model predicted estimated marginal mean differences on MFI fatigue (sub)scales between Anthracycline and non-anthracycline treated patients. Presented with 95% confidence intervals. Abbreviations: SE: Standard error; df: degrees of freedom; Lower.Cl: lower confidence limit; Upper.Cl: upper confidence limit*

|  | Time since onset chemotherapy (months) | Mean difference | Std Error | df | lower.CL | upper.CL | Anthracyclines  (n patients) | No anthracyclines  (n patients) |
| --- | --- | --- | --- | --- | --- | --- | --- | --- |
| EORTC QLQ-C30 fatigue | | | | | | | | |
| Anthracyclines - No anthracyclines | 0 | 0.94 | 2.53 | 5207.7 | -4.03 | 5.90 | 445 | 67 |
| Anthracyclines - No anthracyclines | 0-3 | -0.35 | 2.36 | 4797.0 | -4.99 | 4.28 | 506 | 81 |
| Anthracyclines - No anthracyclines | 3-6 | 2.83 | 2.11 | 3868.1 | -1.30 | 6.96 | 578 | 116 |
| Anthracyclines - No anthracyclines | 6-12 | -2.81 | 1.92 | 3066.5 | -6.57 | 0.96 | 775 | 147 |
| Anthracyclines - No anthracyclines | 12-18 | -3.08 | 2.06 | 3624.0 | -7.12 | 0.96 | 678 | 121 |
| Anthracyclines - No anthracyclines | 18-24 | -3.14 | 2.20 | 4199.6 | -7.45 | 1.18 | 607 | 97 |
| Anthracyclines - No anthracyclines | 24-30 | 0.98 | 2.38 | 4842.2 | -3.69 | 5.64 | 507 | 74 |
| Anthracyclines - No anthracyclines | 30-36 | 3.10 | 2.47 | 5119.3 | -1.75 | 7.95 | 467 | 67 |
| Anthracyclines - No anthracyclines | 36-42 | 0.71 | 2.70 | 5671.4 | -4.59 | 6.00 | 399 | 54 |
| Anthracyclines - No anthracyclines | 42-48 | 7.09 | 3.11 | 6171.7 | 0.99 | 13.20 | 347 | 38 |

*Supplement table 2. Model predicted estimated marginal mean differences on the EORTC QLQ-C30 fatigue symptom scale between Anthracycline and non-anthracycline treated patients. Presented with 95% confidence intervals. Abbreviations: SE: Standard error; df: degrees of freedom; Lower.Cl: lower confidence limit; Upper.Cl: upper confidence limit.*

|  | **Sum Sq** | **Mean Sq** | **DF** | **DenDF** | **F value** | **p value** |
| --- | --- | --- | --- | --- | --- | --- |
| **General fatigue** | | | | | | |
| Time (months) | 1115.385 | 139.423 | 8 | 4660.887 | 16.369 | <0.001 |
| Class of chemotherapy | 18.046 | 18.046 | 1 | 1281.996 | 2.119 | 0.146 |
| Age | 314.555 | 314.555 | 1 | 1179.001 | 36.930 | 0.000 |
| BMI category | 380.643 | 190.321 | 2 | 1134.762 | 22.344 | 0.000 |
| Endocrine treatment | 3.092 | 3.092 | 1 | 1184.209 | 0.363 | 0.547 |
| Time x Class of chemotherapy | 193.286 | 24.161 | 8 | 4660.893 | 2.837 | 0.004 |
| Class of Chemotherapy x Endocrine treatment | 0.103 | 0.103 | 1 | 1183.596 | 0.012 | 0.913 |
| **Physical fatigue** | | | | | | |
| Time (months) | 1682.081 | 210.260 | 8 | 4664.826 | 24.614 | <0.001 |
| Class of chemotherapy | 1.075 | 1.075 | 1 | 1272.847 | 0.126 | 0.723 |
| Age | 59.707 | 59.707 | 1 | 1165.231 | 6.990 | 0.008 |
| BMI category | 749.886 | 374.943 | 2 | 1118.979 | 43.892 | 0.000 |
| Endocrine treatment | 13.309 | 13.309 | 1 | 1171.193 | 1.558 | 0.212 |
| Time x Class of chemotherapy | 244.228 | 30.528 | 8 | 4664.830 | 3.574 | <0.001 |
| Class of Chemotherapy x Endocrine treatment | 0.244 | 0.244 | 1 | 1170.522 | 0.029 | 0.866 |
| **Mental fatigue** | | | | | | |
| Time (months) | 151.695 | 18.962 | 8 | 4647.770 | 2.634 | 0.007 |
| Class of chemotherapy | 0.036 | 0.036 | 1 | 1280.261 | 0.005 | 0.943 |
| Age | 264.036 | 264.036 | 1 | 1183.493 | 36.681 | <0.001 |
| BMI category | 85.528 | 42.764 | 2 | 1141.953 | 5.941 | 0.003 |
| Endocrine treatment | 0.094 | 0.094 | 1 | 1187.870 | 0.013 | 0.909 |
| Time x Class of chemotherapy | 92.269 | 11.534 | 8 | 4647.778 | 1.602 | 0.119 |
| Class of Chemotherapy x Endocrine treatment | 0.313 | 0.313 | 1 | 1187.324 | 0.043 | 0.835 |
| **Reduced activity** | | | | | | |
| Time (months) | 1526.756 | 190.844 | 8 | 4671.940 | 22.660 | <0.001 |
| Class of chemotherapy | 3.013 | 3.013 | 1 | 1279.810 | 0.358 | 0.550 |
| Age | 2.604 | 2.604 | 1 | 1170.974 | 0.309 | 0.578 |
| BMI category | 462.621 | 231.311 | 2 | 1124.181 | 27.465 | <0.001 |
| Endocrine treatment | 16.156 | 16.156 | 1 | 1177.088 | 1.918 | 0.166 |
| Time x Class of chemotherapy | 126.674 | 15.834 | 8 | 4671.944 | 1.880 | 0.059 |
| Class of Chemotherapy x Endocrine treatment | 0.032 | 0.032 | 1 | 1176.405 | 0.004 | 0.951 |
| **Reduced motivation** | | | | | | |
| Time (months) | 393.865 | 49.233 | 8 | 4668.493 | 7.801 | <0.001 |
| Class of chemotherapy | 1.162 | 1.162 | 1 | 1280.610 | 0.184 | 0.668 |
| Age | 47.972 | 47.972 | 1 | 1173.558 | 7.601 | 0.006 |
| BMI category | 180.721 | 90.361 | 2 | 1127.549 | 14.318 | <0.001 |
| Endocrine treatment | 0.425 | 0.425 | 1 | 1179.382 | 0.067 | 0.795 |
| Time x Class of chemotherapy | 56.438 | 7.055 | 8 | 4668.498 | 1.118 | 0.347 |
| Class of Chemotherapy x Endocrine treatment | 1.057 | 1.057 | 1 | 1178.721 | 0.168 | 0.682 |
| **C30 Fatigue symptom scale** | | | | | | |
| Time (months) | 137725.250 | 15302.806 | 9 | 5398.446 | 63.080 | <0.001 |
| Class of chemotherapy | 93.427 | 93.427 | 1 | 1254.610 | 0.385 | 0.535 |
| Age | 5579.849 | 5579.849 | 1 | 1164.805 | 23.001 | <0.001 |
| BMI category | 5457.116 | 2728.558 | 2 | 1121.899 | 11.248 | <0.001 |
| Endocrine treatment | 109.754 | 109.754 | 1 | 1168.966 | 0.452 | 0.501 |
| Time x Class of chemotherapy | 5573.848 | 619.316 | 9 | 5398.367 | 2.553 | 0.006 |
| Class of Chemotherapy x Endocrine treatment | 67.299 | 67.299 | 1 | 1168.171 | 0.277 | 0.599 |

*Supplement table 3. Test statistics for all MFI fatigue scales and EORTC C30 QLQ fatigue symptom scale including interaction between class of chemotherapy and endocrine treatment. Abbreviations: Sum Sq: sum of squares, Mean Sq: mean of the sum of squares; Df: degrees of freedom. DenDF: denominator degrees of freedom.*
